# Clinical implications and considerations for evaluation of in silico algorithms for use with ACMG/AMP clinical variant interpretation guidelines

**DOI:** 10.1186/s13073-017-0508-z

**Published:** 2017-12-18

**Authors:** Lora J. H. Bean, Madhuri R. Hegde

**Affiliations:** 1 Department of Human Genetics, Emory University, Atlanta, GA USA; 2 School of Biological Sciences, Georgia Institute of Technology, Atlanta, GA USA; 3 EGL Genetics, Tucker, GA USA; 4 PerkinElmer Genetics, Pittsburgh, PA USA

**Keywords:** American College of Medical Genetics (ACMG), Association for Molecular Pathology (AMP), Clinical genetics, ClinVar, Diagnostics, In silico algorithm, Variant interpretation

## Abstract

Clinical genetics laboratories have recently adopted guidelines for the interpretation of sequence variants set by the American College of Medical Genetics (ACMG) and Association for Molecular Pathology (AMP). The use of in silico algorithms to predict whether amino acid substitutions result in human disease is inconsistent across clinical laboratories. The clinical genetics community must carefully consider how in silico predictions can be incorporated into variant interpretation in clinical practice.

Please see related Research article: https://doi.org/10.1186/s13059-017-1353-5

## Standardization of variant interpretation

Professional standards and guidelines for clinical interpretation of sequence variants by the American College of Medical Genetics (ACMG) and Association for Molecular Pathology (AMP) [1] are now widely adopted into clinical practice. These guidelines provide a framework for laboratories to evaluate the disease causality of sequence variants in a consistent manner and recommend the classification of these variants into five categories: pathogenic, likely pathogenic, uncertain (variant of uncertain significance; VUS), likely benign, or benign based on the strength of evidence, which is classified into four categories: very strong, strong, moderate, or supporting. There is a direct benefit to patients when a variant is correctly classified as pathogenic or benign, as opposed to a VUS; however, the cost to patients when a classification is incorrect can be an incorrect or a missed diagnosis. The efforts of ACMG/AMP, ClinVar [2] and ClinGen [3] projects, and other attempts to make variant classification freely available [4, 5] has prompted laboratories to compare and harmonize their classification of sequencing variants [6, 7]. These efforts will likely improve clinical care and end the diagnostic odyssey for patients, especially for ultra-rare undiagnosed diseases. A critical tenet of these standards is that variant classification must be dependent upon scientific evidence and weighted according to the type of evidence available, which includes functional studies, segregation studies, comparison of the variant frequency in patients versus the general population, clinical correlation between gene and clinical features of the patient, inferences based on knowledge of the gene or protein structure, in silico predictions, and other evidence detailed in the ACMG/AMP guidelines [1]. Through these newly stimulated community efforts, in silico algorithms that predict whether amino acid substitutions result in human disease are widely, but inconsistently, used by clinical laboratories [6].

## Use of in silico algorithms for clinical interpretation

ACMG/AMP guidelines do not specifically recommend which or how many algorithms to use, but the data can be used as ‘supporting’ evidence for variant interpretation [1]. Many algorithms have been designed to predict the clinical consequences of amino acid substitutions, from the earliest and most prominently used, such as SIFT (sorting intolerant from tolerant) and PolyPhen (polymorphism phenotyping), to the more recently developed methods. There is an active interest in using these methods as evidence for clinical variant interpretation [8, 9]. Ghosh et al. [9] demonstrate an improvement in new methods for in silico variant interpretation, and identify high-performing algorithm combinations that will likely improve accuracy.

Rigorous validation from test design to variant interpretation is required for sequence-based laboratory-developed tests used in genetic diagnostics. Although in silico methods for variant interpretation are freely available, the question of how we should validate these methods given the inherent bias in the datasets available for developing and testing in silico algorithms has not been resolved. These algorithms can be tested only on variants for which the true answer (pathogenic or benign) is known, or at least assumed. However, variants known to be pathogenic or benign have the most evidence (e.g., in vitro assay, disease mechanism, population studies, and segregation studies) to support their reported classification.

Ghosh et al. [9] examined full concordance, lack of concordance, and ‘false concordance’ of pathogenic and benign ClinVar variants among a large number of methods. The reported false concordance rate, depending on the number of algorithms used, ranged from 0.8–5.2% of pathogenic variants assessed as benign, and 10.5–22.5% of benign variants assessed as pathogenic. Should we be willing to accept a method with a high error rate if the evidence carries less weight (considered as ‘supporting’ evidence by ACMG/AMP standards)? Or should we use a method that is more accessible due to cost and time than alternative methods such as functional studies? An error rate of this magnitude would be unacceptable for other types of evidence and would affect how we use this evidence. A study that demonstrates segregation data for a variant associated with disease in a large family with a 5% error rate could not be used for interpretation of the variant because interpretation of these results is heavily dependent on knowing which family members (affected or unaffected) have the variant. Similarly, a 5% error rate, as opposed to the estimated ~ 0.05% false-discovery rate for single nucleotide variants in the Exome Aggregation Consortium (ExAC) dataset [10], could not be used to classify variants as benign based on population frequency. Although large quantities of sequencing data are now publically available [2, 10, 11], the true challenge is interpretation of variants with little or no other information.

Under what circumstances would in silico data provide sufficient evidence to classify a variant as pathogenic or likely benign rather than VUS? The ACMG/AMP guidelines state that substantial supporting or moderate evidence is required to reach a likely pathogenic classification [1]; therefore, in silico supporting data are less likely to sway the final classification. However, in silico supporting data have more potential to impact a likely benign classification because in silico data added to a single piece of strong evidence can achieve a benign classification.

Finally, as in silico methods become more sophisticated we must eventually consider how variants with no clear answer as to whether they are pathogenic or benign will be recognized. Ghosh et al. [9] provide examples of these variants that are likely to produce inconsistent or inaccurate results in in silico predictions: the NP_000234.1:p.Val726Ala *MEFV* variant, which is associated with incomplete penetrance of familial Mediterranean fever, and other variants known to be associated with mild disease or reduced enzyme function, such as NP_000146.2:p.Asn314Asp (the *GALT* Duarte galatosemia allele) or NP_000051.1:p.Asp444His (the *BTD* partial biotinidase deficiency allele). Reports of these variant types have not been addressed in the current version of the ACMG/AMP guidelines. There are likely to be more of these variants discovered for which, even with an abundance of data and careful critical thinking, a community consensus on true clinical consequence is a matter of opinion.

## The future of in silico algorithms to predict variant pathogenicity

In the short term, gene functional assays for variants identified in a clinical laboratory for the purpose of variant classification are not practical, timely, or likely reimbursable. Therefore, the clinical laboratory must maximize other available sources of information. One important resource will be in silico methods to predict variant pathogenicity and the timely question of how we use those methods. Ghosh et al. [9] suggest that different combinations of in silico prediction algorithms will be optimal for classification of pathogenic versus benign sequence variation. However, the combined algorithm approach provides another opportunity for lack of concordance. The question the clinical genetics community must answer is: in our quest for a VUS-free world, how far are we willing to risk clinical care and push variant interpretation in the absence of man, mouse, or experiment?

## Abbreviations

ACMG: American College of Medical Genetics
AMP: Association for Molecular Pathology
VUS: Variant of uncertain significance
